# Severe recurrent hypernatremia and probable lithium-associated nephrogenic diabetes insipidus following pancreatectomy in a patient with schizophrenia: a case report

**DOI:** 10.3389/fmed.2026.1925920

**Published:** 2026-08-27

**Authors:** Yanfei He, Liuyin Jin, Xiaolei Xu, Qing Xia

**Affiliations:** 1 Ningbo No. 2 Hospital, Ningbo, Zhejiang, China; 2 The Second People’s Hospital of Lishui, Lishui, China

**Keywords:** case report, hypernatremia, lithium, nephrogenic diabetes insipidus, perioperative management

## Abstract

**Background:**

Nephrogenic diabetes insipidus (NDI) is characterized by impaired renal responsiveness to arginine vasopressin, causing defective urinary concentration and large volumes of dilute urine. Long-term lithium exposure is an important acquired cause. Perioperative fasting, restricted free-water intake, and ongoing fluid losses may unmask compensated lithium-associated urinary concentrating dysfunction and precipitate severe hypernatremia.

**Case presentation:**

A 61-year-old man underwent laparoscopic resection of a pancreatic tail lesion. His history included schizophrenia, type 2 diabetes mellitus, impaired renal function, long-term lithium therapy, and preoperative polyuria, nocturia, and polydipsia. Preoperative serum sodium was 147.1 mmol/L, creatinine 174.4 μmol/L, and eGFR 35.6 mL/min. Postoperatively, restricted free-water intake was followed by progressive polyuria, persistent negative fluid balance, altered mental status, and worsening renal function. Urine output peaked at 7,900 mL/24 h and serum sodium at 165.5 mmol/L. Despite marked hypernatremia and hypertonicity, urine specific gravity remained ≤1.005. Serum lithium before discontinuation was 0.90 mmol/L. Based on long-term lithium exposure, preoperative symptoms, persistent polyuria, recurrent severe hypernatremia, and low urine specific gravity, probable lithium-associated NDI was considered. Because urine osmolality and a standardized desmopressin response test were unavailable, NDI could not be biochemically confirmed and central diabetes insipidus could not be definitively excluded. Hyperglycemia, gastrointestinal fluid losses, and renal dysfunction may also have contributed.

**Management and outcome:**

Lithium was discontinued after psychiatric and multidisciplinary consultation, and hydrochlorothiazide was initiated. Treatment included gradual free-water replacement, intravenous fluid adjustment, sodium restriction, enteral free water administered separately from enteral nutrition, and close monitoring of serum sodium, potassium, renal function, urine output, glucose, and mental status. The retrospectively estimated free-water deficit was approximately 7.5 L. With combined management, serum sodium, urine output, and consciousness gradually improved. At 12 weeks, serum sodium was 142.3 mmol/L. At 13 months, urine specific gravity remained 1.004, although persistent NDI could not be confirmed.

**Conclusions:**

In patients receiving long-term lithium, postoperative polyuria, recurrent hypernatremia, and low urine specific gravity should prompt consideration of lithium-associated urinary concentrating dysfunction while other causes of water loss are assessed. Careful perioperative medication review, sodium and fluid-balance monitoring, individualized fluid management, and multidisciplinary collaboration are essential.

## 1 Introduction

Hypernatremia is an uncommon electrolyte disorder defined as a serum sodium (sNa) concentration exceeding 145 mmol/L. It reflects a state of hyperosmolality and is occasionally observed among hospitalized patients (1). The reported prevalence of hypernatremia in hospitalized populations ranges from 0.5% to 5.0% (2). If not properly managed, it can lead to fatal outcomes (3). Under physiological conditions, plasma osmolality is maintained within a narrow range of 280 to 295 mOsm/kg through the coordinated actions of arginine vasopressin (AVP), thirst sensation, and renal responsiveness to AVP. Dysfunction in any of these mechanisms may result in hypernatremia, which typically presents with symptoms such as confusion, delirium, increased muscle tone, lethargy, and even coma (4).

Lithium is a classic mood stabilizer used primarily in the treatment of bipolar disorder. Long-term lithium exposure can impair the renal concentrating ability, and some patients may develop nephrogenic diabetes insipidus (NDI), characterized by polyuria and polydipsia. In patients receiving long-term lithium therapy, this concentrating defect may recover only partially after lithium discontinuation (5). Previous studies have reported that lithium-associated NDI or clinically significant impairment of urinary concentrating ability may occur in approximately 20%–40% of patients receiving long-term lithium therapy (6). Such patients are particularly vulnerable to hypernatremia following surgery due to disturbances in water metabolism. If not recognized and treated in a timely manner, this condition may lead to serious neurological complications and even death (7). We report the case of a 61-year-old man with a history of schizophrenia and chronic kidney disease who had been receiving long-term lithium therapy and developed severe hypernatremia after pancreatic surgery. Based on the clinical course and relevant laboratory findings, probable NDI was considered.

## 2 Case presentation

A 61-year-old man was admitted after a pancreatic tail mass had been identified 1 month earlier. His medical history included schizophrenia, type 2 diabetes mellitus, hypertension, cerebral infarction, and coronary artery disease. He had been receiving long-term regular treatment with sustained-release lithium, quetiapine, and clozapine, together with insulin for glycemic control. Further review of his medical history revealed preoperative polyuria, nocturia, and polydipsia. On admission, he was alert, cooperative, able to communicate normally, and able to drink orally. Physical examination showed a temperature of 36.0 °C, pulse rate of 100 beats/min, respiratory rate of 18 breaths/min, and blood pressure of 143/78 mmHg. His height was 1.74 m, body weight was 82 kg, and body mass index was 27.08 kg/m^2^. Skin color was normal, with no edema, rash, or bleeding. There were no obvious signs of dehydration, such as dry oral mucosa, reduced skin turgor, or sunken eyes. Recorded oral water intake was approximately 1,100 mL on hospital day 1 and 1,000 mL on hospital day 2.

**Figure 1:**
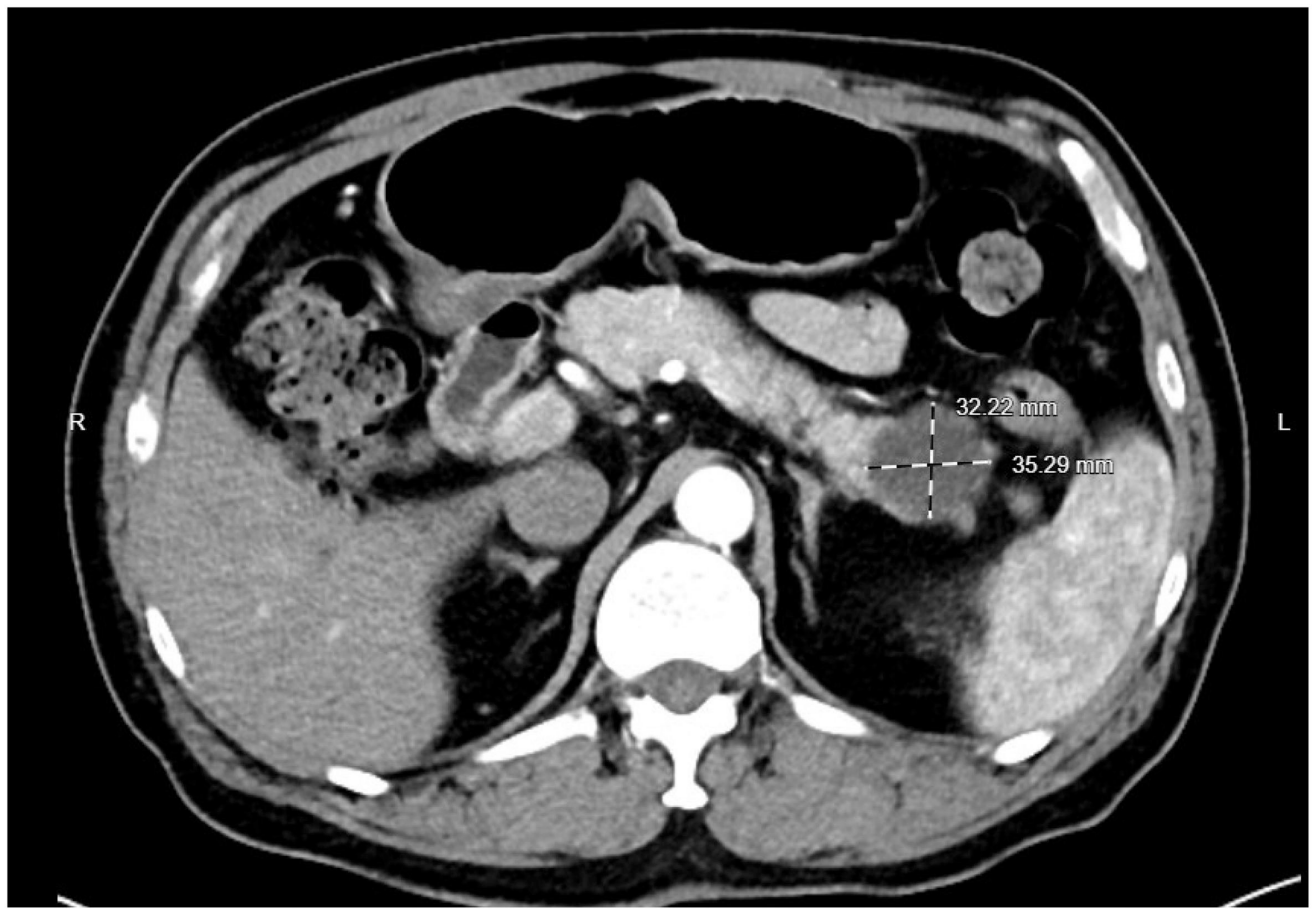
Preoperative contrast-enhanced abdominal computed tomography. Arterial-phase contrast-enhanced abdominal computed tomography obtained on February 15, 2025, showing a cystic lesion in the pancreatic tail measuring approximately 35.3 × 32.2 mm in the axial plane.

Preoperative laboratory testing on February 11, 2025, showed mildly elevated serum sodium at 147.1 mmol/L, serum creatinine of 174.4 μmol/L, and an estimated glomerular filtration rate (eGFR) of 35.6 mL/min, indicating pre-existing renal impairment. The calculated effective plasma osmolality was 314.5 mOsm/L. Contrast-enhanced abdominal computed tomography in the arterial phase on February 15, 2025, demonstrated a cystic lesion in the pancreatic tail measuring approximately 35.3 × 32.2 mm in the axial plane (Figure 1).

**Figure 2:**
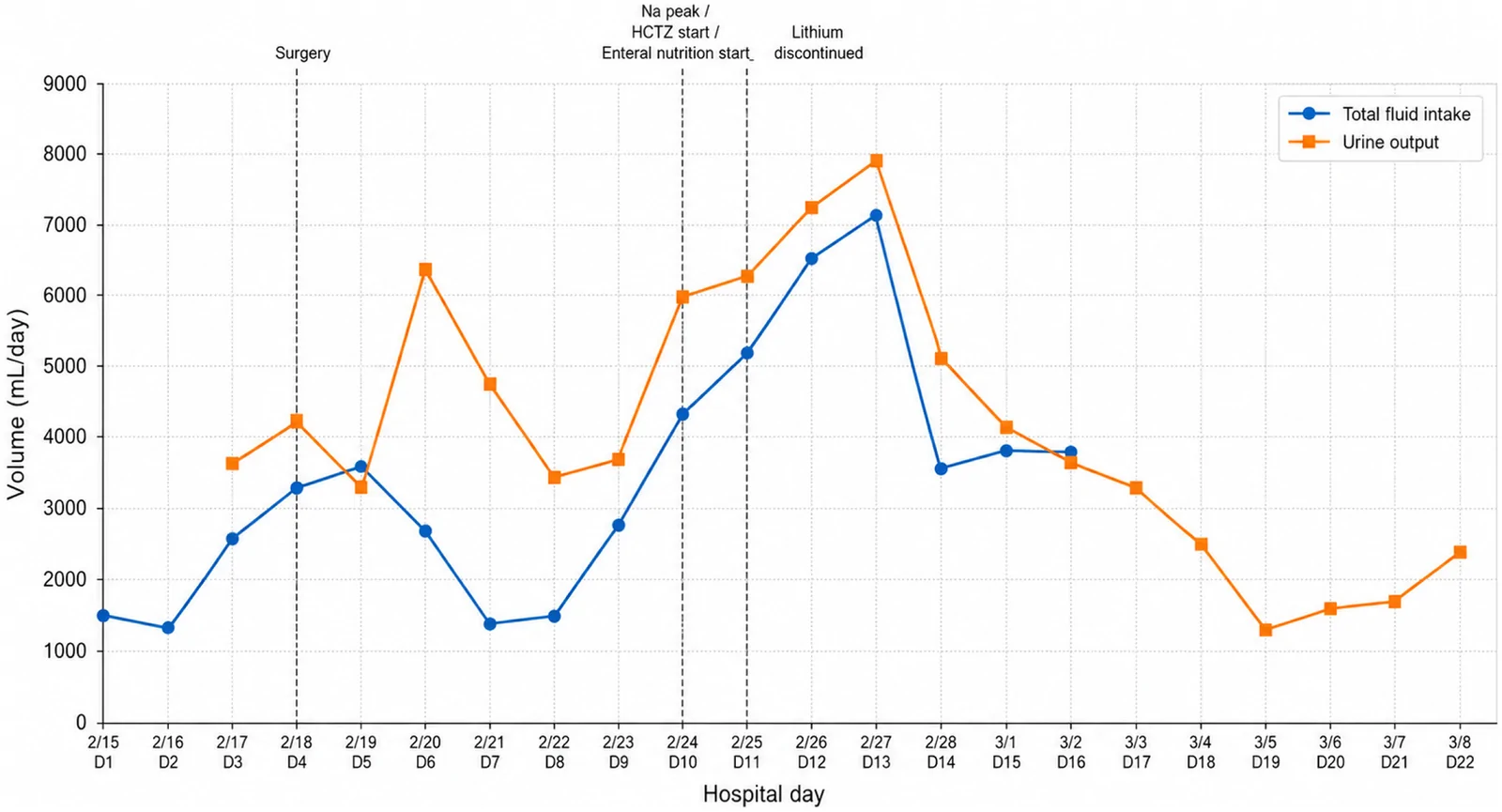
Temporal changes in 24-hour urine output during hospitalization. The patient developed progressive high-volume polyuria after pancreatic surgery. The vertical dashed lines indicate the day of surgery (Day 3), the peak serum sodium level and initiation of hydrochlorothiazide therapy (Day 10), and lithium discontinuation (Day 11). Urine output peaked at 7,900 mL/day on Day 13 and subsequently declined.

On hospital day 3 (February 17, 2025), the patient underwent laparoscopic resection of the pancreatic lesion. He regained consciousness after anesthesia, and his vital signs remained stable during the early postoperative period. Because of postoperative fasting and gastrointestinal recovery, oral free-water intake was substantially restricted. On hospital day 3, no further oral water intake was recorded; total fluid intake was approximately 2,600 mL, whereas 24-hour urine output was 3,600 mL. On hospital day 4, total fluid intake was approximately 3,300 mL, while urine output increased to 4,200 mL, accompanied by a rapid rise in serum sodium to 163.8 mmol/L. Thereafter, progressive high-volume polyuria developed. The 24-hour urine output reached 6,400 mL on hospital day 6 and remained between 6,000 and 7,900 mL/day from hospital days 10 to 13, peaking at 7,900 mL/day before gradually decreasing (Figure 2). Fluid-balance records showed that urine output exceeded total fluid intake on multiple hospital days, indicating recurrent negative fluid balance and inadequate replacement of free-water losses. In addition, the patient experienced postoperative abdominal drainage, gastrointestinal decompression, and diarrhea, resulting in additional nonrenal fluid losses. Therefore, although typical physical signs of dehydration were not continuously documented, the combination of progressive polyuria, persistent negative fluid balance, additional fluid losses, and increasing serum sodium strongly suggested substantial free-water depletion during the hypernatremic period.

**Figure 3:**
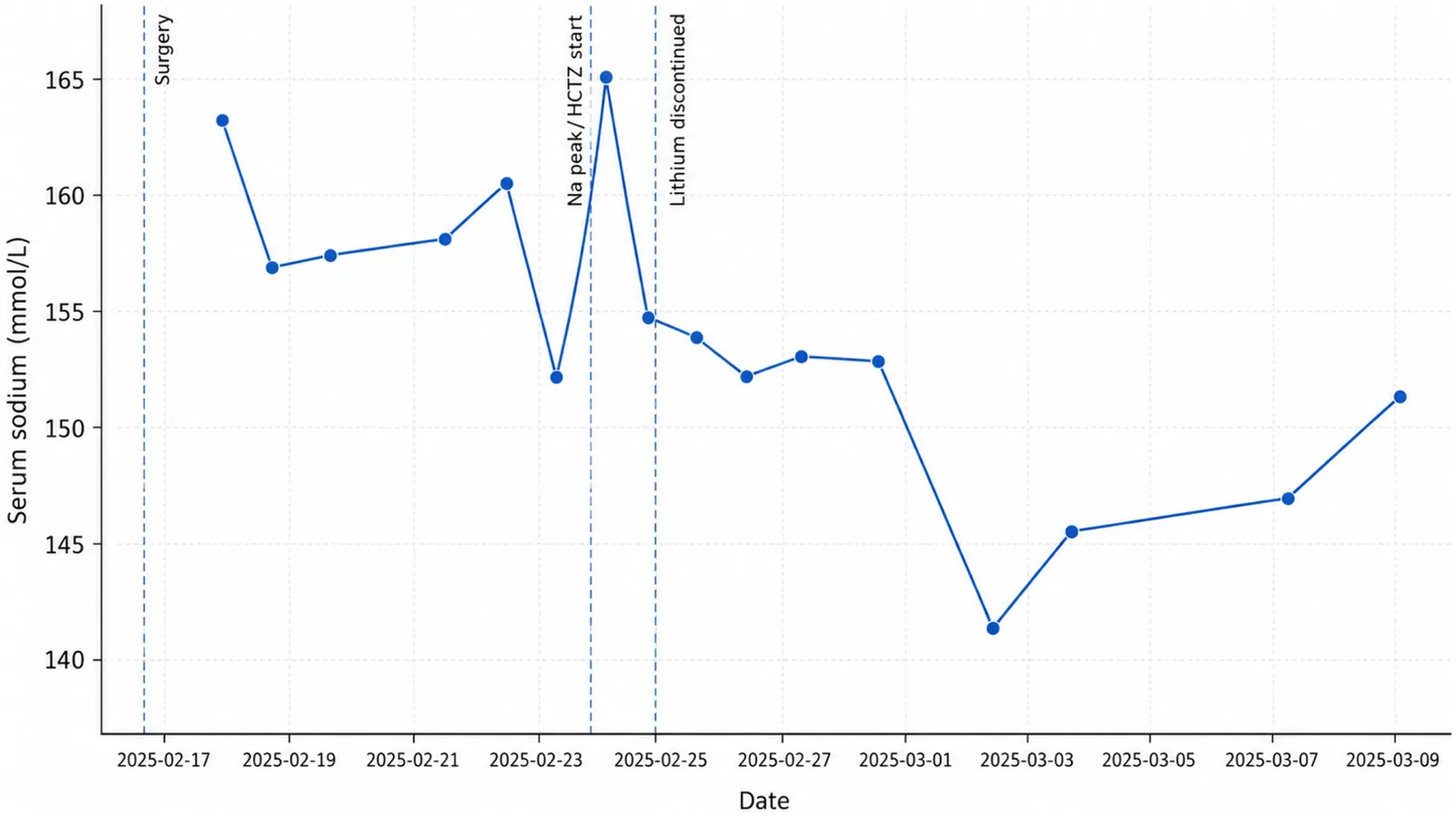
Temporal changes in serum sodium levels and key clinical interventions during hospitalization. Serum sodium increased to a peak of 165.5 mmol/L at 06:04 on February 24, 2025, decreased to 139.8 mmol/L on February 28, and subsequently increased again to 151.6 mmol/L on March 8, demonstrating recurrent hypernatremia. Key clinical interventions, including hydrochlorothiazide initiation and lithium discontinuation, are indicated.

Serum sodium was 163.8 mmol/L on hospital day 4 and reached a peak of 165.5 mmol/L at 06:04 on hospital day 10 (February 24, 2025). It subsequently decreased transiently to 139.8 mmol/L on hospital day 14 but increased again to 151.6 mmol/L on hospital day 22 (March 8, 2025), demonstrating a clearly recurrent pattern of hypernatremia (Figure 3). During the same period, the patient's mental status progressively deteriorated. He was initially alert, but by hospital day 9 he was only able to follow simple commands and could not answer questions clearly. On hospital day 10, when serum sodium reached its peak, he became somnolent. His mental status gradually improved as the water and electrolyte disturbances were corrected, and he was fully alert before discharge.

**Figure 4:**
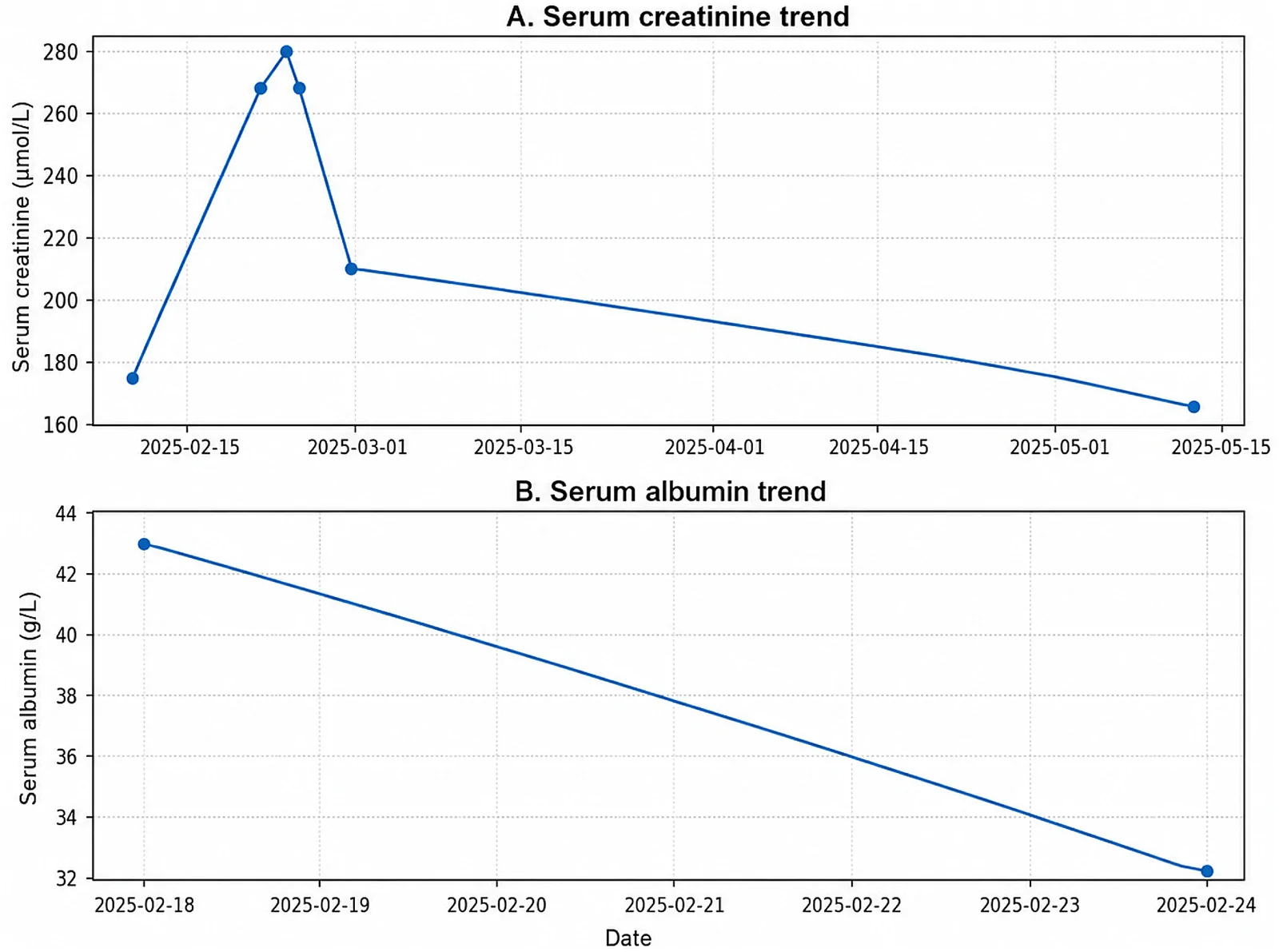
Changes in serum creatinine and serum albumin during hospitalization and follow-up. **(A)** Serum creatinine increased from the preoperative level of 174.4 μmol/L to a peak of 280.3 μmol/L on February 23, 2025, then gradually decreased to 210.0 μmol/L on February 28 and 163.9 μmol/L at approximately 12 weeks after surgery. **(B)** Serum albumin decreased from 43.0 g/L in the early postoperative period to 32.2 g/L on February 24, 2025.

Renal function and nutritional status also deteriorated during this period. Serum albumin decreased from 43.0 g/L in the early postoperative period to 32.2 g/L. Serum creatinine increased from the preoperative value of 174.4 μmol/L to a peak of 280.3 μmol/L on February 23, 2025, with a corresponding eGFR of 20.0 mL/min. By February 28, 2025, serum creatinine had decreased to 210.0 μmol/L. These findings indicated substantial worsening of renal function during the period of severe hypernatremia, followed by gradual recovery (Figure 4).

After further review of the medical records, lithium-associated impairment of urinary concentrating ability was considered an important underlying mechanism contributing to the persistent high-volume polyuria and severe hypernatremia. On February 21, 2025, serum sodium was 156.9 mmol/L and calculated effective plasma osmolality was 337.9 mOsm/L. Later that day, urinalysis showed a urine specific gravity of ≤1.005, while urine glucose was only trace positive (±). The persistence of a low urine specific gravity despite marked hypernatremia and hypertonicity suggested substantially impaired urinary concentrating ability. In the context of long-term lithium exposure, preoperative polyuria, nocturia, and polydipsia, pre-existing renal impairment, persistent postoperative high-volume polyuria, recurrent severe hypernatremia, and low urine specific gravity under hypertonic conditions, probable lithium-associated nephrogenic diabetes insipidus (NDI) was considered one of the most likely underlying diagnoses.

The patient had been taking sustained-release lithium regularly for an extended period and continued to receive lithium throughout the perioperative period. The available records did not indicate a clear period of preoperative discontinuation followed by postoperative reinitiation. At 09:06 on February 25, 2025, a serum lithium concentration obtained before discontinuation was 0.90 mmol/L (laboratory reference range, 0.50–1.50 mmol/L). Lithium was subsequently discontinued following psychiatric consultation and multidisciplinary discussion. Because the available records did not allow reliable reconstruction of the exact long-term daily lithium dose, cumulative duration of therapy, or exact timing of the final preoperative dose, no further inference regarding lithium dose or duration of exposure was made. Although the measured serum lithium concentration was within the laboratory reference range, a single lithium level within the reference range could not exclude chronic lithium-associated impairment of urinary concentrating ability.

Importantly, urine osmolality and quantitative urinary sodium, potassium, and urea measurements were not obtained during the acute phase, and a standardized desmopressin response test was not performed. Therefore, the available evidence was insufficient to establish NDI according to complete biochemical criteria, and central diabetes insipidus could not be definitively excluded. On the basis of the available clinical findings, laboratory data, and medication history, the case was therefore cautiously classified as probable lithium-associated nephrogenic diabetes insipidus, rather than confirmed lithium-induced NDI.

After serum sodium reached 165.5 mmol/L at 06:04 on February 24, 2025, oral hydrochlorothiazide was initiated at 07:58 at a dose of 25 mg three times daily. At 16:31 on the same day, the dose was adjusted to 25 mg twice daily and was continued until discharge. Management of hypernatremia also included restriction of additional sodium intake, administration of low-sodium/hypotonic fluids, and gradual free-water replacement. When clinically feasible, 5% dextrose solution was used as a diluent for intravenous medications.

To further quantify the free-water deficit associated with hypernatremia, a retrospective estimate was calculated from the available data using the formula:

$$\begin{array}{r} \mathrm{Free}\text{-}\mathrm{water}\;\mathrm{deficit}=\mathrm{total}\;\mathrm{body}\;\mathrm{water}\;(\mathrm{TBW})\;\times \\ {}[(\mathrm{measured}\;\mathrm{serum}\;\mathrm{Na}\;/\;\mathrm{target}\;\mathrm{serum}\;\mathrm{Na})-1]. \end{array}$$

For this 61-year-old man, TBW was estimated as 50% of body weight: 0.5 × 82 kg = 41 L. With a measured serum sodium of 165.5 mmol/L and a target serum sodium of 140 mmol/L, the estimated free-water deficit was approximately 7.5 L. It should be emphasized that this value represents a retrospective estimate based on available data rather than a prospectively documented treatment target. Moreover, this calculation did not account for ongoing high-volume urinary losses, diarrhea, gastrointestinal decompression, or other continuing water losses. Therefore, actual free-water requirements needed to be adjusted according to serial fluid-balance and serum sodium measurements.

During the most severe phase of hypernatremia, recorded daily intravenous fluid volumes were approximately 2,800 mL on February 24, 3,000 mL on February 25, 4,000 mL on February 26, 4,000 mL on February 27, and 2,000 mL on February 28, 2025. Fluid therapy was adjusted dynamically according to 24-hour urine output, overall fluid balance, serum sodium, serum potassium, and renal function. Because the exact composition and daily sodium content of some intravenous preparations could not be fully reconstructed from the retrospective medical records, unverified sodium intake was not estimated.

On hospital day 10 (February 24, 2025), because of poor appetite and reduced oral intake, enteral nutrition was initiated through a nasogastric tube at approximately 1,000 mL/day. Enteral nutrition was administered primarily for nutritional support and was recorded separately from enteral free water. Beginning on hospital day 11 (February 25, 2025), approximately 1,000 mL/day of additional free water was administered through the nasogastric tube and subsequently adjusted according to 24-hour urine output, fluid balance, and serial serum sodium measurements. During this period, the patient also underwent gastrointestinal decompression and experienced diarrhea. Thus, in addition to substantial urinary water loss, ongoing nonrenal water losses may have further aggravated the free-water deficit and contributed to the persistence or recurrence of hypernatremia.

Serum sodium was monitored closely during the most critical phase. It was 165.5 mmol/L at 06:04 on February 24, 2025, and decreased to 154.9 mmol/L at 16:56 on the same day. It was 154.2 mmol/L at 06:03 and 152.6 mmol/L at 18:03 on February 25, 153.3 mmol/L at 06:07 on February 26, and 153.1 mmol/L at 06:56 on February 27. Serum sodium subsequently declined further but later increased again, measuring 145.7 mmol/L on March 2, 147.3 mmol/L on March 6, and 151.6 mmol/L on March 8. During the critical period, serum sodium was generally measured once or twice daily, with treatment adjusted according to urine output, renal function, and fluid balance. The available medical records did not document a prospectively predefined target rate of sodium correction. Therefore, only the actual sodium trajectory is reported, and correction rates recommended in guidelines or the literature were not retrospectively presented as predefined treatment targets.

The patient's HbA1c on admission was 6.9%. Reassessment of the temporal relationship between blood glucose and serum sodium showed that severe hypernatremia had already developed before sustained marked hyperglycemia became evident. For example, at 15:26 on February 18, 2025, serum sodium had already increased to 163.8 mmol/L, whereas the blood glucose concentration at a nearby time point was only 7.7 mmol/L. Postoperative hyperglycemia subsequently became more pronounced. On February 24 at 15:55, the same venous biochemical sample showed a blood glucose concentration of 19.53 mmol/L and a serum sodium concentration of 154.9 mmol/L. Using the Katz correction formula, the corrected serum sodium was approximately 158.9 mmol/L, indicating persistent definite hypernatremia even after accounting for hyperglycemia-related transcellular water shifts. Blood glucose increased further later in the clinical course, suggesting that hyperglycemia-associated osmotic diuresis may have contributed additional urinary water loss. Basal and prandial insulin regimens were adjusted according to serial glucose measurements. Because urine osmolality, quantitative urinary glucose, and other urinary solute excretion measurements were unavailable during the acute phase, the relative contributions of osmotic diuresis and impaired urinary concentrating ability to the polyuria could not be accurately quantified.

Following combined management including lithium discontinuation, hydrochlorothiazide therapy, free-water replacement, intravenous fluid management, enteral nutritional support, and intensified glycemic control, serum sodium and urine output gradually decreased overall, mental status recovered, and the patient was discharged after clinical stabilization. Because several therapeutic interventions were implemented within a similar time frame, the clinical improvement could not be attributed to any single treatment measure.

At approximately 12 weeks after surgery (May 13, 2025), serum sodium was 142.3 mmol/L, serum creatinine was 163.9 μmol/L, eGFR was 38.3 mL/min, and calculated effective plasma osmolality was 304.8 mOsm/L. According to the family, daily urine output during April and May 2025 was approximately 2,000–2,500 mL, although standardized 24-hour urine collection was not performed. These findings suggested marked improvement in severe hypernatremia and polyuria compared with the acute phase, although impaired renal function persisted.

Approximately 13 months later, on March 13, 2026, urinalysis continued to show a low urine specific gravity of 1.004, accompanied by urine glucose of 2+. This finding suggested that residual impairment of urinary concentrating ability might still have been present. However, because contemporaneous urine osmolality and standardized 24-hour urine-volume measurements were unavailable, persistent nephrogenic diabetes insipidus could not be confirmed.

## 3 Discussion

The patient developed persistent high-volume polyuria and severe, recurrent hypernatremia after pancreatic surgery, accompanied by markedly impaired urinary concentrating ability. Given the history of long-term lithium exposure, preoperative polyuria, nocturia, and polydipsia, pre-existing renal impairment, and a urine specific gravity that remained ≤1.005 despite marked hypernatremia and hypertonicity, probable lithium-associated NDI was considered the most likely underlying diagnosis. However, because urine osmolality was not measured during the acute phase and a standardized desmopressin response test was not performed, this case should not be regarded as biochemically confirmed lithium-induced NDI.

Lithium carbonate is a classical mood stabilizer widely used in psychiatric disorders. However, its nephrotoxic effects have been well documented, particularly its role in impairing the responsiveness of the distal renal tubules to antidiuretic hormone, thereby disrupting water reabsorption and resulting in nephrogenic diabetes insipidus. Notably, lithium-induced renal damage often has a long latency period, with a median onset of approximately 27 years (8, 9). The principal pathophysiological abnormalities of lithium-associated NDI occur in the renal collecting duct. Aquaporin-2 (AQP2) is a key water channel regulated by arginine vasopressin (AVP) and plays a central role in collecting-duct water reabsorption and urinary concentration. In an animal study, Marples et al. demonstrated that chronic lithium exposure markedly downregulated AQP2 expression in the renal medulla and was accompanied by severe polyuria and impaired urinary concentrating ability. After lithium withdrawal, AQP2 expression recovered only partially, consistent with the slow recovery of lithium-associated urinary concentrating dysfunction observed clinically (10). Subsequent studies have shown that the mechanisms underlying lithium-associated NDI are not limited to reduced AQP2 expression, but also involve abnormal AQP2 trafficking, alterations related to the epithelial sodium channel (ENaC), and remodeling of collecting-duct cell composition and morphology. Together, these molecular and structural abnormalities may impair renal responsiveness to AVP and reduce free-water reabsorption, thereby contributing to persistent polyuria and defective urinary concentration.

Urinary concentrating dysfunction caused by long-term lithium exposure may also persist after treatment discontinuation. Previous studies have shown that, in some patients, the concentrating defect may remain for an extended period even after lithium withdrawal. For chronic lithium-associated nephrotoxicity, some studies suggest that clinically significant renal impairment may develop only after a prolonged latency, potentially over approximately 20–30 years (11). Therefore, in patients with a history of long-term lithium exposure, a serum lithium concentration within the laboratory therapeutic range at a single time point cannot by itself exclude chronic lithium-associated tubular dysfunction.

The present case shares some features with previously reported cases of postoperative lithium-associated NDI but also differs in several important respects. Sze et al. reported in 2006 a patient receiving long-term lithium therapy who developed life-threatening severe hypernatremia after elective spinal surgery because of inadequate oral fluid intake, suggesting that a urinary concentrating defect that had previously been compensated by unrestricted drinking may rapidly decompensate when access to free water is restricted perioperatively (12). In 2019, Shakaroun et al. reported a similar case after esophagectomy. The patient had received lithium therapy for 25 years and had discontinued lithium 10 years earlier, yet developed an increase in serum sodium to 156 mmol/L, increased urine output, and altered mental status after postoperative fasting. NDI was further supported by urine osmolality assessment, water deprivation testing, and desmopressin testing. Hypernatremia subsequently improved with 5% dextrose, enteral free-water replacement, and hydrochlorothiazide (7). Accordingly, the value of the present case does not lie in being the first description of postoperative lithium-associated NDI. Rather, it illustrates a complex clinical course in which latent lithium-associated urinary concentrating dysfunction appeared to decompensate after pancreatic surgery in the setting of restricted free-water intake and was further amplified by postoperative hyperglycemia, gastrointestinal fluid losses, pre-existing renal impairment, and continued lithium exposure. The case also highlights the diagnostic difficulty posed by changes in mental status in a patient with underlying psychiatric disease and demonstrates recurrence of hypernatremia after its initial correction.

Taken together, these cases suggest that lithium-associated urinary concentrating dysfunction may remain relatively compensated while patients retain unrestricted access to water, whereas postoperative fasting or restricted free-water intake may reveal or aggravate the underlying defect. Unlike previously reported cases, the present patient continued lithium therapy throughout the perioperative period and simultaneously had pancreatic surgery, type 2 diabetes mellitus, postoperative hyperglycemia, gastrointestinal decompression, diarrhea, persistent negative fluid balance, and pre-existing renal impairment. Therefore, the severe hypernatremia in this case was more likely multifactorial, reflecting the combined effects of lithium-associated urinary concentrating dysfunction, perioperative free-water deficiency, nonrenal water losses, and later osmotic diuresis rather than a single mechanism.

Notably, severe hypernatremia and high-volume polyuria developed before sustained marked hyperglycemia became evident. Hyperglycemia-related osmotic diuresis therefore could not fully explain the entire clinical course, although it may have further increased urinary water loss later in the hospitalization. Because contemporaneous urine osmolality, quantitative urinary glucose, and other urinary solute excretion measurements were unavailable during the acute phase, the relative contribution of each mechanism could not be quantified accurately. It is therefore more appropriate to interpret this case as postoperative hypernatremia resulting from multiple interacting factors.

Serum sodium increased again to 151.6 mmol/L after the initial decline, indicating that the perioperative disturbance of water homeostasis was persistent and recurrent. Based on a body weight of 82 kg and a peak serum sodium concentration of 165.5 mmol/L, the free-water deficit was retrospectively estimated at approximately 7.5 L using total body water equivalent to approximately 50% of body weight. However, this calculation represents only the static free-water deficit at a single time point and does not include ongoing losses from high-volume urine output, diarrhea, gastrointestinal decompression, or other continuing water losses. It therefore cannot be equated directly with the total volume of fluid replacement required. Management of hypernatremia should account for the pre-existing free-water deficit, ongoing losses, and insensible losses, with free-water and intravenous fluid replacement adjusted dynamically according to serial serum sodium measurements, urine output, fluid balance, and renal function (1, 3). This principle is particularly important in patients such as ours with persistent high-volume polyuria and gastrointestinal fluid losses.

In addition, serum sodium decreased from 165.5 mmol/L at 06:04 on February 24, 2025, to 154.9 mmol/L at 16:56 on the same day, corresponding to a reduction of 10.6 mmol/L over approximately 11 h. Because the original medical records did not document a prospectively predefined target rate of sodium correction, we report only the observed sodium trajectory and do not interpret it as a planned correction rate. For chronic hypernatremia or hypernatremia of uncertain duration, traditional clinical practice generally favors relatively gradual correction, individualized according to the clinical course, neurologic status, ongoing water losses, and serial serum sodium measurements (1). However, evidence defining the optimal correction rate in adults remains limited. A cohort study of critically ill adults found no clear association between more rapid sodium correction and increased risks of mortality, seizures, altered mental status, or cerebral edema, suggesting that traditional correction thresholds should not necessarily be interpreted as absolute limits for all adult patients (13). The recurrence of hypernatremia after its initial correction in the present case further underscores the importance of continued monitoring and dynamic fluid management.

Thiazide diuretics have a paradoxical antidiuretic effect and have been used in the treatment of lithium-associated NDI. Experimental studies have also shown that hydrochlorothiazide can attenuate lithium-associated polyuria and AQP2 downregulation (14). However, urine output did not decrease immediately after hydrochlorothiazide was initiated in this patient. Instead, it continued to increase over the following days, reaching 7,900 mL/day before gradually declining. Accordingly, the subsequent improvement in urine output and serum sodium cannot be attributed to hydrochlorothiazide alone, but more likely reflected the combined effects of lithium discontinuation, free-water replacement, fluid management, glycemic control, and gradual recovery of renal function. Amiloride represents an alternative therapeutic option for lithium-associated NDI because blockade of ENaC can reduce lithium entry into collecting-duct principal cells and thereby mitigate lithium-associated impairment of urinary concentrating ability. Clinical and experimental evidence has supported its use in patients with lithium-associated urinary concentrating dysfunction (15, 16). However, the available medical records did not document the specific rationale for selecting hydrochlorothiazide rather than amiloride in this patient, and this decision therefore cannot be reliably reconstructed retrospectively. In patients with impaired renal function, the use of amiloride would also require careful monitoring of serum potassium and renal function because of the risk of hyperkalemia.

In this case, the early postoperative changes in mental status were difficult to distinguish from manifestations potentially related to the patient's underlying psychiatric disorder, illustrating the diagnostic complexity of patients with multiple chronic conditions. However, the patient simultaneously had severe hypernatremia, marked polyuria, and persistent negative fluid balance, and his mental status improved as the water and electrolyte disturbances were corrected. Metabolic and electrolyte abnormalities were therefore likely important contributors to the episode of altered consciousness. In patients with pre-existing psychiatric disorders, attributing new changes in consciousness or behavior solely to the underlying psychiatric condition may delay recognition of severe hypernatremia and urinary concentrating dysfunction. This case therefore also emphasizes the importance of timely collaboration among psychiatry, endocrinology, nephrology, and surgical teams.

Perioperative lithium management represents another important clinical lesson from this case. The patient continued lithium therapy throughout the perioperative period until it was discontinued following psychiatric and multidisciplinary consultation after the development of marked polyuria and severe hypernatremia. We do not retrospectively interpret this as evidence of a perioperative medication error. Rather, the clinical course highlights the importance of systematically assessing long-term lithium therapy before surgery. The UK Clinical Pharmacy Association Handbook of Perioperative Medicines recommends preoperative assessment of urea and electrolytes, thyroid function, and electrocardiography in patients receiving lithium; for major elective surgery, lithium discontinuation 24 h before surgery is recommended. Postoperatively, lithium should be restarted only when renal function is stable, with close electrolyte monitoring and maintenance of adequate hydration (17). Reviews of perioperative psychotropic medication management have likewise emphasized the potential interactions between psychotropic agents and anesthetic drugs and their broader perioperative implications (18). Therefore, in patients receiving long-term lithium therapy who are scheduled for major surgery, preoperative assessment should not be limited to a single serum lithium measurement. A history of polyuria, nocturia, and polydipsia should be actively sought, together with assessment of serum sodium, renal function, and possible urinary concentrating dysfunction. Postoperatively, if patients are unable to maintain adequate free-water intake because of fasting, altered mental status, or delayed gastrointestinal recovery, urine output, fluid balance, serum sodium, blood glucose, and renal function should be monitored closely. In patients with long-term lithium exposure who develop unexplained high-volume polyuria and progressive hypernatremia, lithium-associated NDI should be considered early, with urine osmolality and, when appropriate, dynamic diagnostic testing used to further clarify the underlying cause.

This case report has several limitations. First, because this was a retrospective single-case report, several key diagnostic data were unavailable. Urine osmolality and quantitative urinary sodium, potassium, and urea measurements were not obtained during the acute phase, and a standardized desmopressin response test was not performed. Therefore, despite supportive findings including long-term lithium exposure, preoperative polyuria, nocturia, and polydipsia, persistent high-volume polyuria, recurrent severe hypernatremia, and urine specific gravity ≤1.005 under hypertonic conditions, the available evidence was insufficient to establish NDI according to complete biochemical criteria, and central diabetes insipidus could not be definitively excluded. Accordingly, this case was classified only as probable lithium-associated NDI. Second, postoperative polyuria and hypernatremia occurred in a clearly multifactorial setting. Postoperative fasting and restricted free-water intake, persistent negative fluid balance, gastrointestinal decompression, diarrhea, worsening renal function, and osmotic diuresis related to marked hyperglycemia later in the clinical course may all have contributed to the development, persistence, and recurrence of hypernatremia. Because contemporaneous urine osmolality, quantitative urinary glucose, and other urinary solute excretion measurements were unavailable, the relative contributions of lithium-associated urinary concentrating dysfunction, osmotic diuresis, and nonrenal water losses could not be quantified accurately. Consequently, the entire episode of polyuria and hypernatremia cannot be attributed solely to lithium-associated NDI. Third, some details of lithium exposure could not be reconstructed reliably from the retrospective records. Although long-term lithium therapy and continuation throughout the perioperative period until discontinuation after psychiatric and multidisciplinary consultation could be confirmed, the exact long-term daily dose, cumulative duration of treatment, and timing of the final dose could not be determined reliably. Therefore, the relationship between lithium dose or duration of exposure and the severity of urinary concentrating dysfunction could not be assessed. The serum lithium concentration measured before discontinuation was 0.90 mmol/L; however, a single value within the laboratory reference range cannot establish a causal relationship between long-term lithium exposure and chronic tubular dysfunction. Fourth, the patient had pre-existing renal impairment, but earlier stable prehospital serum creatinine and eGFR values were unavailable, and therefore a well-established long-term renal baseline could not be determined. Serum creatinine increased from the available preoperative value of 174.4 μmol/L to a peak of 280.3 μmol/L postoperatively. Although this represented an increase to approximately 1.61 times the preoperative value, the available creatinine measurements were not sufficiently frequent to establish whether the increase occurred within the 48-hour or 7-day time windows specified by the KDIGO criteria for acute kidney injury (AKI). In addition, the patient had marked polyuria rather than oliguria. Therefore, the available data were insufficient to reliably confirm or stage AKI according to KDIGO criteria, and we describe the renal-function change more cautiously as acute perioperative worsening of renal function on a background of pre-existing renal impairment. Postoperatively, the patient also experienced substantial negative fluid balance, severe hypernatremia, hyperglycemia, gastrointestinal fluid losses, and perioperative physiological stress. The observed deterioration in renal function was therefore likely multifactorial and cannot be attributed solely to long-term lithium exposure or lithium-associated NDI. In addition, tubular injury biomarkers such as NGAL and KIM-1 were not measured, precluding direct assessment of the extent of possible lithium-associated tubular injury. Fifth, although major fluid intake, urine output, enteral nutrition, and part of the free-water supplementation during hospitalization could be reconstructed, the exact composition and sodium content of some intravenous fluids, as well as the complete free-water and sodium balance during hypernatremia treatment, could not be determined retrospectively. Prospective calculation of the free-water deficit and a predefined target sodium correction rate were also not fully documented. Therefore, the independent effects of individual fluid-management strategies on serum sodium could not be quantified rigorously. Similarly, hydrochlorothiazide was initiated after serum sodium reached its peak, but lithium discontinuation, free-water replacement, fluid management, glycemic control, and renal recovery occurred within overlapping time periods. The subsequent improvements in urine output and serum sodium therefore cannot be attributed to hydrochlorothiazide alone. The medical records also did not document the specific clinical rationale for selecting hydrochlorothiazide rather than amiloride. Sixth, although follow-up data at approximately 12 weeks and 13 months after surgery were added and showed overall improvement in severe hypernatremia and marked polyuria, standardized 24-hour urine-volume measurements, urine osmolality, and comprehensive assessment of urinary concentrating function were unavailable during long-term follow-up. Therefore, it remains uncertain whether urinary concentrating ability recovered completely, and the long-term reversibility of potential lithium-associated tubular dysfunction cannot be determined accurately. Finally, this report describes a single patient whose clinical course was influenced by psychiatric disease, type 2 diabetes mellitus, pre-existing renal impairment, pancreatic surgery, and multiple perioperative interventions. The generalizability of the findings and the ability to draw causal inferences are therefore limited. Further case reports or larger studies incorporating complete urine osmolality and urinary solute assessments, detailed lithium exposure histories, standardized diagnostic testing, and long-term follow-up of tubular function are needed to better characterize the diagnosis, management, and long-term outcomes of perioperative lithium-associated urinary concentrating dysfunction.

## 4 Conclusion

Postoperative hypernatremia may result from multiple interacting factors, and underlying urinary concentrating dysfunction deserves particular attention in patients receiving long-term lithium therapy who also have pre-existing renal impairment. This case suggests that probable lithium-associated NDI should be considered in the differential diagnosis when persistent high-volume polyuria, progressive or recurrent hypernatremia, and low urine specific gravity develop after surgery. At the same time, other contributors, including inadequate free-water intake, hyperglycemia-related osmotic diuresis, gastrointestinal fluid losses, and changes in renal function, should be carefully evaluated.

Early recognition of disordered water homeostasis, systematic review of perioperative medications, serial monitoring of serum sodium, urine output, and fluid balance, and individualized free-water and fluid management according to ongoing losses are important for reducing the risk of complications associated with severe hypernatremia. In patients with long-term lithium exposure, preoperative assessment of polyuria, nocturia, polydipsia, and renal function, together with close multidisciplinary collaboration among psychiatry, endocrinology, nephrology, and surgical teams, may facilitate earlier recognition and management of perioperative disturbances in water homeostasis.

## Data Availability

The original contributions presented in the study are included in the article/Supplementary Material, further inquiries can be directed to the corresponding author.
